# Bayesian hierarchical network autocorrelation models for estimating direct and indirect effects of peer hospitals on outcomes of hospitalized patients

**DOI:** 10.1007/s41109-024-00627-1

**Published:** 2024-06-14

**Authors:** Guanqing Chen, A. James O’Malley

**Affiliations:** 1 Department of Anesthesia, Critical Care and Pain Medicine, Beth Israel Deaconess Medical Center, Harvard Medical School, Boston, MA 02215 USA; 2 Department of Biomedical Data Science , Geisel School of Medicine at Dartmouth, Lebanon, NH 03756 USA; 3 The Dartmouth Institute for Health Policy and Clinical Practice, Geisel School of Medicine at Dartmouth, Lebanon, NH 03756 USA

**Keywords:** Bayesian inference, Direct and indirect peer effects, Diffusion of robotic surgery, Hierarchical network autocorrelation model

## Abstract

When an hypothesized peer effect (also termed social influence or contagion) is believed to act between units (e.g., hospitals) above the level at which data is observed (e.g., patients), a network autocorrelation model may be embedded within a hierarchical data structure thereby formulating the peer effect as a dependency between latent variables. In such a situation, a patient’s own hospital can be thought of as a mediator between the effects of peer hospitals and their outcome. However, as in mediation analyses, there may be interest in allowing the effects of peer units to directly impact patients of other units. To accommodate these possibilities, we develop two hierarchical network autocorrelation models that allow for direct and indirect peer effects between hospitals when modeling individual outcomes of the patients cared for at the hospitals. A Bayesian approach is used for model estimation while a simulation study assesses the performance of the models and sensitivity of results to different prior distributions. We construct a United States New England region patient-sharing hospital network and apply newly developed Bayesian hierarchical models to study the diffusion of robotic surgery and hospital peer effects in patient outcomes using a cohort of United States Medicare beneficiaries in 2016 and 2017. The comparative fit of models to the data is assessed using Deviance information criteria tailored to hierarchical models that include peer effects as latent variables.

**Supplementary Information:**

The online version contains supplementary material available at 10.1007/s41109-024-00627-1.

## Introduction

The network autocorrelation model (NAM) involves the study of relationships among social units and their interdependent behaviors (O’Malley and Marsden 2008). For instance, as described in Doreian (1980) and Friedkin (1990), a classic linear NAM assuming that a peer effect acts on the outcomes themselves is specified as: $Y=\rho W Y+X \beta +\varepsilon$, $\varepsilon \sim N\left( 0, \sigma ^{2} I\right)$, where *Y* is a vector of outcomes, *W* is a matrix whose elements represent social ties between actors, *X* is a matrix of covariates, $\varepsilon$ is the error term and $\rho$ quantifies the direct peer effect between subjects. Doreian (1980) also describes an alternative model in which the error term rather than the outcome variable is interdependent: $Y=X \beta +\varepsilon$, with $\varepsilon =$ $\rho W \varepsilon +\vartheta$. This model is also well known as a simultaneously autoregressive (SAR) model. Furthermore, Friedkin (1990) introduces a model including both interdependent exogenous and endogenous forms of social influence: $Y=\rho _{1} W_{1} Y+\rho _{2} W_{2} Z+X \beta +\varepsilon$, where *Z* is a column vector for an exogenous variable. However, in the current literature on social network analysis, none of these models examined the interdependence among the actors when they are at a higher level in a hierarchical data structure than the units on which observations are made. Such a scenario may arise in a study of peer effects among hospitals in which the goal is to determine whether patient outcomes are directly impacted by peer hospitals. Meeting such a goal is important as knowing whether a hospital’s adoption of a technology impacts the outcomes of a greater population of patients than just their own patients is important for policy-makers to know in order to make decisions regarding the priority of different incentive programs aiming to improve the quality of patient care and outcomes.

In Chen and O’Malley (2024), we delved into the research conducted by Dong and Harris (2015) on hierarchical spatial autoregressive models (HSAR) that account for hierarchical spatial data structures involving geographic units. Compared to network data, spatial data typically has a simpler typology in which distances between points or areas are compliant with the triangle inequality. Additionally, in this framework, the peers of geographic units can only exert indirect influence on individuals in the focal geographic unit through spatial connections at the geographic unit level as opposed to directly impacting individuals in the focal geographic unit.

In this paper, we first develop the basic hierarchical network autocorrelation model by adapting the HSAR in Dong and Harris (2015) to social network data assuming the peer effects of actors (e.g., hospitals) higher in the hierarchical structure than the level at which observations are made (e.g., patients). Second, we develop a novel extended hierarchical network autocorrelation model that includes an extra parameter to allow direct inter-level influence of hospitals on patients of other hospitals. This extended model relaxes the “no direct effect” restriction of the basic HSAR model in which peer hospitals may indirectly impact the patients from the focal hospital through their impact on the focal hospital (i.e., an indirect effect of peer hospitals) but does not allow peer hospitals to directly impact patients of the focal hospital. The basic HSAR model can be considered a special case of the extended model in which direct impact is not allowed. We study the mean and variance of the observation-level outcomes as a function of these two network autocorrelation parameters to gain insights into the mechanisms that they represent. The adaptation of HSAR to social network data has not been studied in the literature to date while the extension of the model to allow for direct (across-level spillover) effects is an entirely new topic.

Due to the complexity of the hierarchical network structure, we complete a Bayesian specification of the model and use Bayesian computational methods to fit each of our hierarchical network autocorrelation models. We perform a series of simulation studies to quantify the properties and demonstrate the performance of Bayesian posterior median estimators of the model parameters including the autocorrelation parameters under different prior distributions; the sensitivity of posterior inferences to the prior distribution assumed for the focal peer effect parameter $\rho$ is of particular interest. To alleviate concerns with commonly-used uniform priors for $\rho$, we develop a new prior that imposes uniformity on a natural transformation of $\rho$.

Our study has three main methodological contributions. First, we develop two hierarchical network autocorrelation models assuming the peer effects of actors operate at a higher level of the model than the observation level. Further, we allow for direct and indirect across-level peer effects of actors. Third, to explore and interpret the peer effects, we assess the functional dependence of the marginal mean and variance of the outcomes on the network parameters, density and covariates, compare the two models in terms of model fit and results for the robotic surgery application, develop novel priors for the model parameters, including for a transformed uniform prior distribution for $\rho$ designed to be both a non-informative prior but also to aid model estimation, and explore the sensitivity of posterior inferences to the prior distribution for $\rho$. This paper extends our paper (Chen and O’Malley 2024) published in the Proceedings of the Complex Networks 2023 Conference. We further explore the impact of higher level covariates of both the focal actor and the peer actors on observation level outcomes in section "Notation and models", thereby extending our models to inter-level spillover effect models; demonstrate our Bayesian estimation approach in greater detail with the development and derivation of prior distributions for the model parameters and the corresponding posterior distributions in section "Bayesian hierarchical network autocorrelation model and estimation"; assess the performance of our Bayesian estimation approach using a weighted network whose distribution of edge weights is matched to that in our empirical robotic surgery example using simulations and compare the ensuing findings to those under the binary-valued network in section "Simulation study"; and describe the construction of the patient-sharing hospital network used in the robotic surgery example in section "The impact on patient quality of hospitals’ adoption of robotic surgery".

Our motivating example is an observational study in which the objective is to understand the full impact of the adoption of robotic surgery on the time to discharge from hospital of patients undergoing prostatectomy surgery. Robotic surgery, as a robotically-assisted and minimally-invasive procedure, is commonly used in prostatectomy for prostate cancer and also assists in the treatment of lung cancer, kidney cancer and colorectal cancer (Lee 2009; Chandra et al. 2015; Mirnezami et al. 2010; Novellis et al. 2017). Several advantages of robotic surgery such as shorter hospital stays, less pain, and lower risk of infection have previously been discussed (Barbash and Glied 2010). Using the 2016 United States (US) fee-for-service Medicare claims data, we construct a US New England region hospital network for patients with prostate, lung, kidney and colorectal cancer. We estimate the peer effects among hospitals on prostatectomy time to discharge post-surgery of US Medicare patients in 2017 to allow a lagged peer effect of network interdependency and to partially protect inferences against reverse causality.

Although the development of two models for analyzing peer effects when the data are hierarchically-structured is the methodological focus and primary contribution of this paper, the findings from applying our models to the robotic surgery network and hospital attribute data will potentially assist policy-makers wanting to provide incentives to hospitals to adopt new medical technologies that are beneficial to patients. In particular, the novel extended hierarchical network autocorrelation model will provide insights into whether a hospital’s adoption of technologies generally benefits patients in a local area (e.g., by improving general standards of surgical quality including strengthening infection control measures in emergency rooms) such that patients who receive surgeries at other hospitals also benefit.

## Notation and models

Throughout this paper, the term “ego” refers to the focal actor being studied while the term “alter” refers to the actors connected to the ego in a network, also referred to as “peers”.

### Hierarchical network autocorrelation model

In our adaptation of the HSAR in Dong and Harris (2015), we first assume that peer-effects only act on individual subjects generating observations through their impact on the cluster-effects of the units (network actors) in which the individuals are grouped, such as in the following model:

1 $$\begin{aligned} &Y=Z \theta +B \delta +\varepsilon \\ &\delta =\rho W \delta +X \beta +\tau \end{aligned}$$

where $\varepsilon \sim N\left( 0, \sigma ^{2} I_{N}\right)$, $\tau \sim N\left( 0, \omega ^{2} I_{g}\right)$, *Y* is a vector of length *N* containing the values of a response variable for *N* observations, *Z* is a $N \times k$ matrix for *k* observation level covariates whose first column is a vector of $1_{s}$ corresponding to the intercept parameter, *X* is a $g \times l$ matrix for *l* cluster level covariates, $\delta$ is a vector of length *g* representing the random effect of *g* network actors and *B* is a $N \times g$ matrix linking the random effect $\delta$ back to *Y*. In addition, $\varepsilon$ and $\tau$ represent the errors at the observational and cluster levels and *W* is a $g \times g$ matrix quantifying the relationships between the actors in the associated network. The $i j^{\text{ th } }$ entry of *W*, $W_{i j}$, represents the relationship of actor *i* to actor *j*.

The matrix *W* is constrained to be a non-negative row-normalized matrix, reflecting the non-existence of negative influences and that relative exposures are the conduit through which social influence transmits. The diagonal of *W* consists of zeros as self-ties are not permitted in the network. The focal parameter $\rho$ is the peer effect corresponding to the indirect effect of alters on the outcomes of individuals in the role of the ego.

Letting $A=I_{g}-\rho W$, to ensure *A* is non-singular and its determinant, $|A| \ne 0$, the range of $\rho$ needs to be restricted. Following Anselin (1988) and LeSage (2000), we restrict the parameter space of $\rho$ to $\left( 1 / \lambda _{\min }, 1 / \lambda _{\max }\right)$, where $\lambda _{\max }$ and $\lambda _{\min }$ are the maximum and minimum eigenvalues of the row-normalized *W*. For a row-normalized *W*, $1 / \lambda _{\max }=1$ and $1 / \lambda _{\min } \le -1$ (Stewart 2009) with the value of $1 / \lambda _{\min }$ becoming more negative with increasing network density. Network density equals $M /(g(g-1))$ for directed networks and $2 M /(g(g-1))$ for undirected networks, where $\textrm{M}$ is the observed number of ties and $g(g-1)$ is the number of possible directed ties.

We compute the marginal mean and variance of *Y* to explore and interpret the peer effect. If *A* is non-singular, the marginal mean and variance satisfy:

$$\begin{aligned} & E(Y)=Z \theta +B A^{-1} X \beta \\ &{\text {var}}(Y)=B A^{-1} \omega ^{2} I_{g} A^{-1^{T}} B^{T}+\sigma ^{2} I_{N} \end{aligned}$$

Applying the Neumann series, when the norm $|\rho W|<1$ it follows that:

$$\begin{aligned} A^{-1}=I_{g}+(\rho W)+(\rho W)^{2}+\cdots +(\rho W)^{N}+\cdots =\sum _{h=0}^{\infty }(\rho W)^{h} \end{aligned}$$

The above demonstrates that both the marginal mean and variance of the model depend on $\rho$ and that $A^{-1}$ is an infinite-order polynomial function of $\rho$ and *W*.

### Extended hierarchical network autocorrelation model

**Fig. 1:**
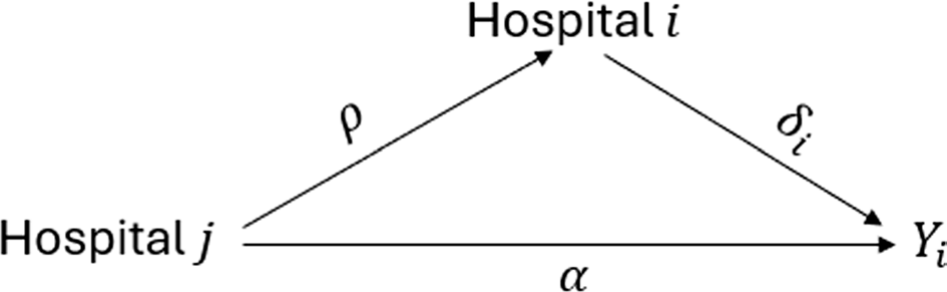
Directed acyclic graph for direct and indirect peer effect

A restriction on the model in (1) is that conditional on $\delta _{i}$ and $\delta _{j}$ there is no direct dependence between the vector of observations $Y_i=(Y_{1i}, \ldots, Y_{mi})^{T}$ and $Y_j=(Y_{1j}, \ldots, Y_{nj})^{T}$, where *n* and *m* denote the number of patients within hospital *i* and *j* respectively, for any $i \ne j$. However, in practice, hospitals may directly influence the patients of other hospitals. For example, if the patients of peer hospitals benefit from improved quality of care at their hospitals, they may incentivise better health behaviors and thus outcomes in the patients of the ego hospital. In general, a peer-hospital may impact patients indirectly via hospital-to-hospital transmission of peer influence that filters down to patients or through a second mechanism that acts directly from a peer hospital through their own patients to the ego hospital’s patients. To allow for the possibility of a direct peer-hospital to ego-hospital-patient effect, we introduce an extended hierarchical network autocorrelation model with an extra parameter quantifying direct across-level influence of hospitals on patients of other hospitals:

2 $$\begin{aligned} &Y=Z \theta +B\left[ \delta +\alpha W_{1} \delta \right] +\varepsilon \\ &\delta =\rho W_{2} \delta +X \beta +\tau \end{aligned}$$

where $\varepsilon \sim N\left( 0, \sigma ^{2} I_{N}\right)$, $\tau \sim N\left( 0, \omega ^{2} I_{g}\right)$, and $\alpha$ is an unrestricted parameter that quantifies the direct network effect of alters on the outcome of individuals from the ego. Intuitively, the parameters $\rho$ and $\alpha$ have an analogy to indirect and direct effects in a mediation analysis, although they are not necessarily on the same scale. Under (2), $\rho$ is the indirect effect of peer hospital on the outcomes of patients of the ego hospital through their impact on that hospital’s performance while $\alpha$ is the direct effect that acts independently of the ego hospital (see Fig. 1).

The matrices $W_{1}$ and $W_{2}$ in (2) could represent different types of relationships between actors; e.g., $W_{1}$ could be built on geographic distances between hospitals while $W_{2}$ could be built on patient-sharing information between hospitals. With only a single source of network relationship information, in our study we set $W_{1}=W_{2}=W$. Model (1) is the special case of model (2) in which $\alpha =0$.

Letting $G=B\left[ I_{g}+\alpha W\right]$, we compute the marginal mean and variance of *Y* under (2):

$$\begin{aligned} &E(Y)=Z \theta +G A^{-1} X \beta \\ &{\text {var}}(Y)=G A^{-1} \omega ^{2} I_{g} A^{-1^{T}} G^{T}+\sigma ^{2} I_{N} \end{aligned}$$

To help interpret $\alpha$ and $\rho$ and differentiate the behavior and properties of the model in (2) from those of the model in (1), as now described in section "Illustration of marginal mean and variance of extended model with simulated data" we numerically evaluated these expressions across a range of values of $\alpha$ and $\rho$ and visualized the results.

### Illustration of marginal mean and variance of extended model with simulated data

**Fig. 2:**
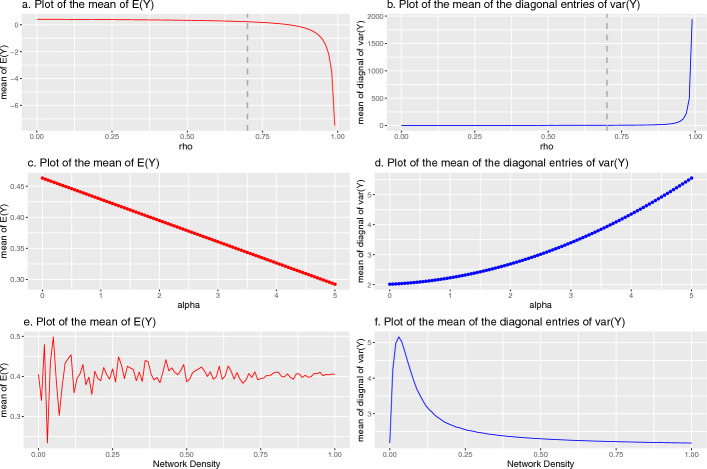
The marginal mean and variance of the model along with the change of $\rho$, $\alpha$ and network density. Note: The vertical dashed line at $\rho = 0.7$ denotes an upper bound peer-effects are unlikely to exceed in practice

To gain insight into the effects captured by $\rho$ and $\alpha$ under the model in (2), we simulated 100 datasets under this model assuming a network containing 50 hospitals and 30 individuals per hospital (model (1) is a special case of model (2) where $\alpha =0$ making it sufficient to only consider model (2) in the simulation). To determine how the marginal mean and variance of the model change with increasing $\rho$, we plot the average of the mean of the elements of *E*(*Y*) and the average of the diagonal elements of ${\text {var}}(Y)$ over 100 drawn values on the vertical-axis against $\rho$ on the horizontal-axis (Fig. 2a and b, noting that values of $\rho >0.7$ are unlikely in practice). Similarly, we evaluate the relationship between $\alpha$ and the marginal mean and variance of the model (Fig. 2c and d). Finally, we investigate the association between the network density *d* and the marginal mean and variance of the model (Fig. 2e and f).

Figure 2a and b shows that the magnitude of the marginal average mean and variance of *Y* increases when the value of $\rho$ increases and accelerates exponentially upwards when $\rho$ approaches its upper bound of 1. When $\rho$ approaches 1, the determinant of *A* approaches zero and the entries of $A^{-1}$ become increasingly large leading to extreme exponential behavior. Similar results are found for negative values of $\rho$. Figure 2c reveals a linear decreasing association between the marginal average mean of *Y* and $\alpha$ while Fig. 2d shows that the corresponding marginal variance increases with $\alpha$. From Fig. 2e and f, we find that the marginal mean and variance display volatile behavior when the network density is smaller than 0.1. When the network density is small, e.g., $d<0.1$, the simulated network often contains isolated nodes. To overcome computational issues in matrix row-normalization that occur with isolated nodes, our specification of *W* assumes that isolates are equally influenced by all other actors. Accordingly, the volatile behavior of the marginal mean and variance of *Y* as density approaches 0 is due to the rapid escalation in the prevalence of isolates.

**Fig. 3:**
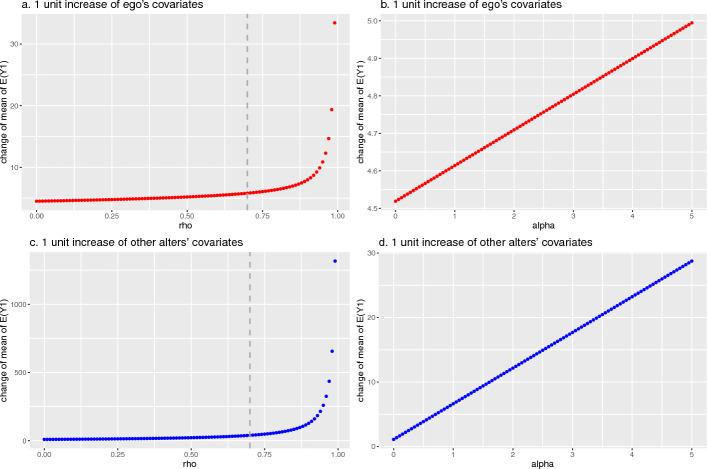
The change of the marginal mean of the model following a 1 unit increase of ego’s and alters’ covariates across a range of values of $\rho$ and $\alpha$. Note: The vertical dashed line at $\rho = 0.7$ denotes an upper bound peer-effects are unlikely to exceed in practice

A special case of our models is the spillover effects model in which an individual’s outcome is influenced by their alters’ covariates. Therefore, to illustrate how the ego and alter covariates associate with the outcome, we compute and plot the change in the expected value of the mean of $E\left( Y_{.j}\right)$ for subject *j* under (i) a 1 unit increase in a covariate $X_j$ (i.e., the change $X_{j} \rightarrow X_{j}+1$) and (ii) a 1 unit increase in the mean of subject *j*’s alters covariates $X_{-j}$ (i.e., the change $X_{-j} \rightarrow X_{-j}+1$). The corresponding changes of the *j*th ego’s mean are $E\left( Y_{.j} \mid X_{j}+1\right) -E\left( Y_{.j} \mid X_{j}\right)$ and $E\left( Y_{.j} \mid X_{-j}+1\right) -E\left( Y_{.j} \mid X_{-j}\right)$, respectively. Analogous simulations under the two scenarios were conducted with respect to $\rho$ and $\alpha$ (see Fig. 3a and b for $\rho$ and $\alpha$, respectively, when $X_{j} \rightarrow X_{j}+1$ and likewise Fig. 3c and d when $X_{-j} \rightarrow X_{-j}+1$). For illustration we consider the case when $j=1$, the individuals within hospital 1.

Figure 3a shows that the average value of $E\left( Y_{.1}\right)$ due to a 1 unit increase in the ego’s covariate (i.e., $X_{1} \rightarrow X_{1}+1$) increases with increasing values of $\rho$ and resembles an exponential trend as $\rho$ approaches its upper bound. Similarly, in Fig. 3c, the average values of the mean of $E\left( Y_{.1}\right)$ due to a 1 unit change in the weighted average of the alters’ covariates (i.e., $X_{-1} \rightarrow X_{-1}+1$) increases with increasing values of $\rho$ and follows an exponential trend as $\rho$ approaches its upper boundary. Although these two figures depict similar exponential behavior, the magnitude of the changes differ as evinced by the differing scales on the vertical axes. Exponential behavior occurs because the determinant of *A* is close to zero and the elements of $A^{-1}$ become increasingly large when $\rho$ approaches its upper bound. Additionally, in Fig. 3b and d, we find that the change of average values of mean of $E\left( Y_{.1}\right)$ due to the ego’s own covariate values and that of their peers increases are linear with increasing $\alpha$ but differ in the scale of the changes.

## Bayesian hierarchical network autocorrelation model and estimation

We adapt estimation approaches for linear NAMs to our hierarchical NAMs. A simulation study of Dittrich et al. (2017) shows that Bayesian modeling and estimation outperforms maximum likelihood estimation with respect to bias and the level and width of interval estimators (termed credible intervals in Bayesian statistics). To account for the complexity of the hierarchical network structure, in our analysis we use a full Bayesian specification of the model and Bayesian computational methods.

A Bayesian analysis relies on specification of a prior distribution, a likelihood function, and the derivation of the posterior distribution. The prior distribution, $p(\varphi )$ contains the prior information or beliefs for the model parameters $\varphi$. The likelihood function $f(y \mid \varphi )$ summarizes the information in the data. By Bayes rule, the posterior distribution satisfies: $p(\varphi \mid y) \propto p(y \mid \varphi )$ $p(\varphi )$. Bayesian point and interval estimates may be derived from the posterior distribution using direct Monte Carlo (or closed-form) probability evaluations.

### Prior distributions for Bayesian modelling

Despite the importance of prior distributions in Bayesian analysis, there has been little study of the properties of different prior distributions for $\rho$ within the hierarchical network autocorrelation model framework.

We have an interest in developing non-informative priors for $\rho$ that enable stable computation of statistical inferences by pulling estimators away from boundary values while introducing minimal information into the analysis. As discussed in Chen and O’Malley (2024), we propose 3 prior distributions for $\rho$ with different ranges and shapes to investigate the sensitivity of the posterior distribution to the prior for $\rho$. These priors include: (1) A uniform prior $p(\rho ) \propto 1$ over the range $1 / \lambda _{\min }<\rho <1 / \lambda _{\max }$ (as previously discussed, $1 / \lambda _{\max }=1$ and $1 / \lambda _{\min }$ become much smaller than $-1$ when network density increases). (2) A uniform prior for $\rho$ with support $(-1,1)$, a symmetric and more restricted parameter space that matches that of correlation coefficients such as the Pearson and Spearman’s rank correlation coefficients. (3) As discussed in Chen and O’Malley (2024), an improper uniform prior on a parameter equal to the following transformation of $\rho$ to the entire real line:

$$\begin{aligned} g(\rho )=\log \left( \frac{1 / \lambda _{\max }-\rho }{\rho -1 / \lambda _{\min }}\right) = \log \left( \frac{1-\rho \lambda _{\max }}{\rho \lambda _{\min } - 1}\frac{\lambda _{\min }}{\lambda _{\max }} \right) \end{aligned}$$

The above expression corresponds to the generalized logit function. Therefore, the implied prior for $\rho$ is given by:

$$\begin{aligned} p(\rho ) \propto \frac{1}{\left( 1 / \lambda _{\max }-\rho \right) \left( \rho -1 / \lambda _{\min }\right) } = \frac{\lambda _{\max }\lambda _{\min }}{(1 - \rho \lambda _{\max })(\rho \lambda _{\min }-1)} \end{aligned}$$

having positive support for $\rho \in \left( 1 / \lambda _{\min }, 1 / \lambda _{\max }\right)$. Our transformed uniform prior emulates Jeffery’s prior applied to classic linear NAMs (Dittrich et al. 2017).

In addition, we consider several informative priors including the normal prior for $\rho$ given by $p(\rho ) \sim N\left( 0.36, 0.7^{2}\right)$ used in Dittrich et al. (2017) in the context of linear NAMs, which we refer to as the normal informative prior. We evaluate the performance of this normal prior in our simulation experiment along with other priors above. In addition, because positive values of $\rho$ are more common than negative values of $\rho$ in practice, we also analyze the performance of the positive uniform (0, 1) prior for $\rho$ in our simulation study in section "Simulation study" when the true value of $\rho$ is non-negative.

**Fig. 4:**
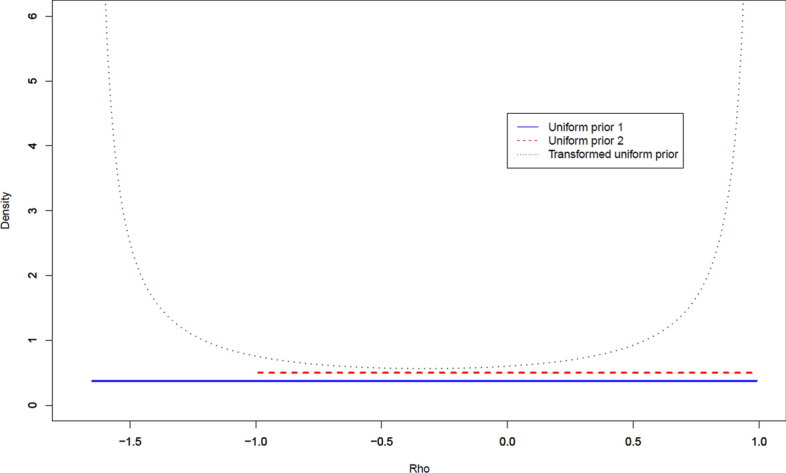
Prior distributions of $\rho$. Note: Uniform prior 1 is $p(\rho ) \propto 1$ over the range $1 / \lambda _{\min }<\rho <1 / \lambda _{\max }$ while uniform prior 2 is $p(\rho ) \propto 1$ over the range $-1<\rho <1$

For further illustration and comparisons, we use the network constructed in our motivating robotic surgery example with $1 / \lambda _{\max }=1$ and $1 / \lambda _{\min }=-1.660$ to plot the density function of our proposed three priors for $\rho$. As shown in Fig. 4, the transformed uniform prior is “U-shaped” and has more prior mass near to its boundary values $\left\{ 1 / \lambda _{\min }, 1 / \lambda _{\max }\right\}$ than under the two uniform priors of $\rho$.

To complete a Bayesian specification of the model, we assign improper flat priors on $\sigma$ and $\omega$; i.e., $\left( \sigma ^{2}, \omega ^{2}\right) \propto 1/(\sigma \omega )$. Alternatively, a half Cauchy prior can be placed on $\omega$ to take advantage of its desirable properties for hierarchical models (Gelman 2006). Because the development of a half Cauchy prior for $\omega$ in relation to NAMs has not been discussed in the literature, we present the derivation in the “Appendix”. The results using the half Cauchy prior are similar to those for the uniform prior on $\omega$ in our analysis (see simulation results in section "Simulation study"). We specify the flat prior $p(\theta, \beta ) \propto 1$ for $(\theta,\beta )$, although indistinguishable results are found from assigning normal priors centered at 0 with large variances (“non-informative normal priors”) for $\theta$ and $\beta$. In addition, for the model in (2), we assign a flat prior $p(\alpha ) \propto 1$ to $\alpha$ with no restriction on its range. Because $\alpha$ is structurally analogous to the regression coefficients $\beta$ and $\theta$, the same non-informative priors that are used for $\beta$ and $\theta$ may also be used for $\alpha$. For instance, one may also consider using a normal distribution with a large variance as a prior for $\alpha$.

### Bayesian modelling

Under model (1), the likelihood function is given by:

$$\begin{aligned} f\left( Y \mid \theta, \delta, \sigma ^{2}\right) =\left( 2 \pi \sigma ^{2}\right) ^{-N / 2} \exp \left( -\frac{(Y-Z \theta -B \delta )^{T}(Y-Z \theta -B \delta )}{2 \sigma ^{2}}\right), \end{aligned}$$

and the conditional prior distribution of $\delta$ as:

$$\begin{aligned} p\left( \delta \mid \beta, \omega ^{2}, \rho \right) =|A|\left( 2 \pi \omega ^{2}\right) ^{-\frac{g}{2}} \exp \left( -\frac{(A \delta -X \beta )^{T}(A \delta -X \beta )}{2 \omega ^{2}}\right) \end{aligned}$$

Due to the complexity of the model and the large number of parameters, the joint posterior distribution is non-standard and direct sampling from it is intractable. Therefore, we use a hybrid Gibbs-sampling Metropolis-Hastings algorithm that sequentially draws from the conditional posterior distribution of each parameter given the data and current values of all other parameters (Geman and Geman 1984).

As seen in the probability distributions functions presented below, due to conjugacy the conditional posterior distributions for each of $\sigma ^{2}, \omega ^{2}, \beta, \theta$ and $\delta$ have well-known closed-forms:

$$\begin{aligned} & P\left( \sigma ^{2} \mid \delta, \theta, \beta, \omega ^{2}, \rho, Y\right) \sim I G\left( \frac{N-1}{2}, \frac{(Y-Z \theta -B \delta )^{T}(Y-Z \theta -B \delta )}{2}\right) \\ &P\left( \omega ^{2} \mid \delta, \theta, \beta, \sigma ^{2}, \rho, Y\right) \sim I G\left( \frac{g-1}{2}, \frac{(A \delta -X \beta )^{T}(A \delta -X \beta )}{2}\right) \\ &P\left( \beta \mid \delta, \sigma ^{2}, \rho, \omega ^{2}, \theta, Y\right) \sim N\left( \left( X^{T} X\right) ^{-1} X^{T} A \delta,\left( X^{T} X\right) ^{-1} \omega ^{2}\right) \\ & P\left( \theta \mid \delta, \sigma ^{2}, \rho, \omega ^{2}, \beta, Y\right) \sim N\left( \left( Z^{T} Z\right) ^{-1} Z^{T}(Y-B \delta ),\left( Z^{T} Z\right) ^{-1} \sigma ^{2}\right) \\ & p\left( \delta \mid \beta, \omega ^{2}, \rho, \sigma ^{2}, \theta, Y\right) \\ &\quad \sim N\left( \left( \frac{\sigma }{\omega } A^{T} \frac{\sigma }{\omega } A+B^{T} B\right) ^{-1}\left( \left( \frac{\sigma }{\omega } X \beta \right) ^{T} \frac{\sigma }{\omega } A+(Y-Z \theta )^{T} B\right) ^{T}, \sigma ^{2}\left( \frac{\sigma }{\omega } A^{T} \frac{\sigma }{\omega } A\right. \right. \\ &\quad \left. \left. +B^{T} B\right) ^{-1}\right) \\ \end{aligned}$$

making sampling from them straightforward. In contrast, the conditional posterior of $\rho$:

3 $$\begin{aligned} p\left( \rho \mid \beta, \omega ^{2}, \delta, \sigma ^{2}, \theta, Y\right) \propto |A| \exp \left( -\frac{(A \delta -X \beta )^{T}(A \delta -X \beta )}{2 \omega ^{2}}\right) p(\rho ) \end{aligned}$$

does not have a form conducive for direct sampling. Therefore, we approximate (3) and use a Metropolis Hastings algorithm with an independent candidate generating function that functions like accept-reject sampling and when feasible allows more efficient exploration of the posterior distribution than a random-walk candidate generating distribution (Dittrich et al. 2017). As demonstrated in the derivation in the “Appendix”, the resulting candidate generating distribution of $\rho$ when $p(\rho ) \propto 1$, $1 / \lambda _{\min }<\rho <1 / \lambda _{\max }$, is the truncated normal distribution (*TN*):

4 $$\begin{aligned} &p\left( \rho \mid \beta, \omega ^{2}, \delta, \sigma ^{2}, \theta, Y\right) \sim TN\left( \mu ^{*}, V^{*}\right) \text{ for } 1 / \lambda _{\min }<\rho <1 / \lambda _{\max } {\text{ with }} \\ &\mu ^{*} =\frac{\delta ^{T} W^{T}(\delta -X \beta )}{\omega ^{2} \sum \lambda _{i}{ }^{2}+\delta ^{T} W^{T} W \delta } \\ &V^{*} =\frac{\omega ^{2}}{\omega ^{2} \sum \lambda _{i}{ }^{2}+\delta ^{T} W^{T} W \delta } \end{aligned}$$

where $\lambda _{i}$ for $i=1, \ldots, g$ are the eigenvalues of *W*. Under $p(\rho ) \propto 1$, $-1<\rho <1$, the implied candidate generating distribution of $\rho$ is $TN\left( \mu ^{*}, V^{*}\right)$ with support $-1<\rho <1$. For the transformed uniform prior of $\rho$, we use the same candidate generating distribution in Eq. (4) to sample $\rho$.

We use the same prior distributions and MCMC sampling procedure as for the model in (1) to fully specify and fit the model in (2). The conditional posterior of $\alpha$ is then:

$$\begin{aligned} p\left( \alpha \mid \beta, \omega ^{2}, \rho, \sigma ^{2}, \theta, Y, \delta \right) \sim N\left( \frac{\delta ^{T} W^{T} B^{T} K}{\delta ^{T} W^{T} B^{T} B W \delta }, \frac{\sigma ^{2}}{\delta ^{T} W^{T} B^{T} B W \delta }\right), \end{aligned}$$

where $K=Y-Z \theta -B \delta$. The derivation of the conditional posteriors of all other parameters of the model in (2) are presented in the “Appendix”.

## Simulation study

As discussed in Chen and O’Malley (2024), we conducted a simulation study involving 1,500 hypothetical patients receiving care from 50 hospitals (30 patients per hospital) in a hospital network to evaluate the performance of the models in (1) and (2) under different priors for $\rho$. Specifically, we generated undirected binary-valued network matrices representing whether peer hospitals share patients or not with network density (*d*) $= 0.2, 0.4, 0.6, 0.8$ using the R package “sna”. In each case, the resulting adjacency matrix was row-normalized to form the matrix *W*. We considered the following values of $\rho =-0.5,-0.2,0,0.2,0.5$ and $\alpha =-2,2$. For each model, three patient-level covariates plus an intercept and three hospital-level covariates were included with the elements of the matrices *Z* and *X* consisting of random draws from the standard normal distribution. The true values of $\sigma ^{2}$ and $\omega ^{2}$ were set to 1. For each scenario, we generated 500 simulated datasets and for each simulation dataset drew 20,000–50,000 samples from the joint posterior distribution. We used the posterior median as the Bayesian point estimator because the conditional posterior distributions of $\rho$ tends to be skewed, in which case the posterior median is often a better measure of the center of the distribution than the posterior mean (Dittrich et al. 2017). In our study, the posterior distributions of $\rho$ and $\alpha$ are close to symmetric resulting in the posterior mean, median and mode estimators of $\rho$ and $\alpha$ yielding similar results. The bias of the posterior median estimator of $\rho$ was computed by evaluating $bias_{\rho }=\frac{1}{500}\sum _{s=1}^{500}\left( \hat{\rho }_{s}-\rho _{true}\right)$, where *s* is the simulation counter, $\hat{\rho }_{s}$ is the posterior median of $\rho$ in simulated dataset *s*, and $\rho _{true}$ is the value of $\rho$ used in generating each of the 500 simulated datasets. We similarly calculated the frequentist mean squared error (MSE) of $\hat{\rho }_{s}$ as $MSE_{\rho }=\frac{1}{500}\sum _{s=1}^{500}\left( \hat{\rho }_{s}-\rho _{true}\right) ^{2}$. Finally, we computed the coverage rate of the $95 \%$ equal-tailed credible interval for $\rho$ by evaluating $coverage_{\rho }=\frac{1}{500}\sum _{s=1}^{500} I\left( \rho _{true} \in CI_{\rho s}\right)$, where *I*(*e*) is the indicator function equal to 1 if the event *e* is true and 0 otherwise, and $CI_{\rho s}=(q_{\rho, s,0.025},q_{\rho, s,0.975})$ is the equal-tailed $95\%$ credible interval of $\rho$ in simulated dataset *s* in which $q_{\rho, s,1-\kappa }$ is the $1-\kappa$ quantile of the posterior distribution for $\rho$ in simulated dataset *s*. We estimate $CI_{\rho s}$ non-parametrically by extracting the $2.5 \%$ and $97.5 \%$ smallest to largest ordered values in the retained sample of 500 posterior draws of $\rho$ in the analysis of dataset *s*. We used the same approach to compute the bias, MSE and coverage rate of $\alpha$.

The simulations reveal that our Bayesian estimation approach performs well with respect to bias, MSE and the coverage rate of the $95\%$ equal-tailed credible intervals of $\rho$ and $\alpha$ (for detailed results, see Tables S1 to S6 in the Supplementary document of the manuscript). The bias of $\rho$ and $\alpha$ in our hierarchical models increase with increasing network density, consistent with findings regarding bias of $\rho$ for linear NAMs reported and discussed in Mizruchi and Neuman (2008) and Dittrich et al. (2017). By comparing the performance of the three different priors for $\rho$, we found that the range of the prior has more impact than its shape on the estimated values. In particular, due to the asymmetric support of $\rho$, $1 / \lambda _{\min }<\rho <1 / \lambda _{\max }$, the full-range uniform prior for $\rho$ yields a posterior median estimator of $\rho$ exhibiting an asymmetric bias pattern either side of 0. In contrast, bias is much more symmetric around 0 under the uniform $(-1,1)$ prior for $\rho$, especially when network density is large. For example, for model (1) with the uniform $(1 / \lambda _{\min }, 1 / \lambda _{\max })$ prior for $\rho$, when $d=0.8$, bias $=0.025$ if $\rho =-0.5$ and bias $=-0.298$ if $\rho =0.5$. Under the uniform $(-1,1)$ prior for $\rho$, when $d=0.8$ and $\rho =-0.5$ we obtain bias $=0.241$ while if $\rho =0.5$ then bias $=-0.274$. These results imply that as network density increases in a binary-valued network, it becomes more challenging for the model to identify $\rho$. Intuitively, as density increases the information in the data about $\rho$ declines due to the vast number of connections in the binary valued network making the variation across the actors in the extent to which they are more or less connected with other actors much lower than when density is low. It is under this high density scenario that slight differences in the non-informative prior specification for $\rho$ nontrivially impact the resulting posterior distribution.

We also conducted a simulation study to evaluate the performance of model (1) and (2) for the motivating example in section "The impact on patient quality of hospitals’ adoption of robotic surgery" by building an undirected weighted network whose network density and distribution of edge weights are similar to the network in the motivating example (45 hospitals and 1,306 patients with network density $=0.779$ and the mean and standard deviation of the weighted edge $\approx$ 226 and 664, respectively). In the simulated data sets, we set the number of hospitals to 50 with 30 individuals per hospital (the total number of individuals, $N=1{,}500$), and the network density to 0.8. We then generated edge-weights from the ${\text {Gamma}}(0.1,2000)$ distribution. Other settings were maintained as for the simulations in Chen and O’Malley (2024). The results for model (1) and (2) under the various priors described in section "Prior distributions for Bayesian modelling" are shown in Tables 1, 2, 3 and 4, respectively.

Compared to the binary-valued network, we observed smaller bias and MSE of the estimators of $\rho$ and $\alpha$ under the weighted network. In addition, we found bias is more symmetric around 0 under the uniform $(1 / \lambda _{\min }, 1 / \lambda _{\max })$ prior (because $1 / \lambda _{\min }$ is close to $-1$, some results are identical for the Unif 1 and Unif 2 priors). For example, in Table 3, using the uniform prior $p(\rho ) \propto 1$ over $1 / \lambda _{\min }<\rho<$ $1 / \lambda _{\max }$, the bias of $\rho$ is $-0.001$ when $\rho =-0.2$ and is $-0.013$ when $\rho =0.2$, which emulate the results obtained when $p(\rho ) \propto 1$ over $-1<\rho <1$. This is because: (1) *W* based on the weighted edge network may contain more information about relationships between the actors in a network than the binary-valued network; (2) Under these simulation settings, $1 / \lambda _{\min }$ is close to $-1$ and $1 / \lambda _{\max } =1$; therefore, the interval support for the uniform prior $(1 / \lambda _{\min }, 1 / \lambda _{\max })$ and the transformed uniform prior is more symmetric.

The results from using the normal informative prior $N(0.36, 0.7^2)$ are very similar to those under the original three priors of $\rho$ described earlier while the results from using the half-Cauchy prior for $\omega$ are very similar to those under the uniform prior for $\omega$. Because 0 is at the boundary of the parameter space of (0, 1), as expected the positive uniform prior *U*(0, 1) yielded a large bias and zero coverage rate when $\rho =0$ under both model (1) and (2).

**Table 1:** Bias, mean squared error (MSE), and 95% coverage rates (Rate) of $\rho$ using uniform priors (Unif 1 for $1 / \lambda _{\min }<\rho<$ $1 / \lambda _{\max }$ and Unif 2 for $-1<\rho <1$) and the transformed uniform prior (T Unif) for model (1)

	$\rho =-0.2$			$\rho =0$			$\rho =0.2$		
	Unif 1	Unif 2	T Unif	Unif 1	Unif 2	T Unif	Unif 1	Unif 2	T Unif
$$\rho$$									
Bias	−0.002	−0.002	−0.003	−0.007	−0.007	−0.004	−0.012	−0.012	−0.004
MSE	0.011	0.011	0.012	0.012	0.012	0.012	0.012	0.012	0.012
Rate	0.972	0.972	0.970	0.968	0.968	0.966	0.962	0.962	0.962

For each value of $\rho$, the results represent the bias, MSE and Rate of $\rho$. The results are rounded to 3 decimal places

**Table 2:** Bias, mean squared error (MSE), and 95% coverage rates (Rate) of $\rho$ using uniform (0, 1) prior (Pos Unif), $N(0.36, 0.7^2)$ prior (Norm) for $\rho$ and half Cauchy prior (HC) for $\omega$ for model (1)

	$\rho =0$			$\rho =0.2$			$\rho =0.5$		
	Pos Unif	Norm	HC	Pos Unif	Norm	HC	Pos Unif	Norm	HC
$$\rho$$									
Bias	0.089	0.006	0.002	0.017	−0.005	−0.028	−0.005	−0.017	−0.061
MSE	0.011	0.012	0.040	0.009	0.012	0.042	0.011	0.011	0.041
Rate	0	0.952	0.964	0.978	0.956	0.954	0.954	0.946	0.946

For each value of $\rho$, the results represent the bias, MSE and Rate of $\rho$. For the half Cauchy scenario, we use uniform prior $1 / \lambda _{\min }<\rho<$ $1 / \lambda _{\max }$ for $\rho$. The results are rounded to 3 decimal places

**Table 3:** Bias, mean squared error (MSE), and 95% coverage rates (Rate) of $\rho$ using uniform priors (Unif 1 for $1 / \lambda _{\min }<\rho<$ $1 / \lambda _{\max }$ and Unif 2 for $-1<\rho <1$) and transformed uniform prior (T Unif) and assume an improper flat prior for $\alpha$ in model (2)

	$\rho = -0.2$			$\rho =0$			$\rho =0.2$		
	Unif 1	Unif 2	T Unif	Unif 1	Unif 2	T Unif	Unif 1	Unif 2	T Unif
$$\rho$$									
Bias	−0.001	−0.003	−0.001	−0.007	−0.006	−0.002	−0.013	−0.013	−0.002
MSE	0.017	0.017	0.018	0.018	0.018	0.019	0.018	0.018	0.019
Rate	0.962	0.968	0.958	0.956	0.956	0.95	0.946	0.946	0.944
$$\alpha$$									
Bias	−0.006	−0.004	−0.006	−0.006	−0.007	−0.006	−0.007	−0.007	−0.007
MSE	0.003	0.003	0.003	0.003	0.003	0.003	0.003	0.003	0.003
Rate	0.936	0.924	0.936	0.94	0.934	0.94	0.936	0.936	0.934

For each value of $\rho$ in the first row, the results represent the bias, MSE and Rate of $\rho$ and $\alpha$. The results are rounded to 3 decimal places

**Table 4:** Bias, mean squared error (MSE), and 95% coverage rates (Rate) of $\rho$ using the uniform (0, 1) prior (Pos Unif), $N(0.36, 0.7^2)$ prior (Norm) for $\rho$ and half Cauchy prior (HC) for $\omega$ and assume an improper flat prior for $\alpha$ in model (2)

	$\rho =0$			$\rho =0.2$			$\rho =0.5$		
	Pos Unif	Norm	HC	Pos Unif	Norm	HC	Pos Unif	Norm	HC
$$\rho$$									
Bias	0.108	0.007	0.004	0.026	−0.006	−0.038	−0.023	−0.016	−0.064
MSE	0.016	0.016	0.044	0.012	0.017	0.045	0.015	0.014	0.039
Rate	0	0.964	0.956	0.97	0.95	0.956	0.948	0.946	0.95
$$\alpha$$									
Bias	0.007	−0.006	0	−0.001	−0.007	0.001	0.002	0.003	−0.001
MSE	0.003	0.003	0.003	0.004	0.003	0.003	0.004	0.004	0.003
Rate	0.942	0.936	0.93	0.94	0.934	0.958	0.956	0.946	0.944

For each value of $\rho$ in the first row, the results represent the bias, MSE and Rate of $\rho$ and $\alpha$. For the half Cauchy scenario, we use uniform prior $1 / \lambda _{\min }<\rho<$ $1 / \lambda _{\max }$ for $\rho$. The results are rounded to 3 decimal places

## The impact on patient quality of hospitals’ adoption of robotic surgery

Robotic surgery, with its advantages of shorter hospital stays, less pain and faster recovery, has been widely used on patients suffering from many health conditions, particularly in the cure of prostate cancer, lung cancer, kidney cancer and colorectal cancer. In our study, we are interested in whether the extent to which peer hospital adoption of robotic surgery influences the time from when a patient undergoes prostatectomy surgery until they are discharged at a hospital. We first construct a “New England region” of the six states in the Northeastern US (Maine, New Hampshire, Vermont, Massachusetts, Connecticut, and Rhode Island) patient sharing hospital network. As a substantial sub-region of the US, the New England region provides an adequate sample size for our analysis. Consequently, analyses focused on the New England region are considered to be based on a sufficient magnitude and richness of data to be informative and have a realistic chance of detecting effects of clinical significance. Following the approach introduced in Moen et al. (2016) and O’Malley et al. (2020), for each pair of physicians we compute the weighted edges between physicians by summing the geometric means of the number of visits the same patient made to each physician in the pair across all patients suffering one of these four cancer types. To clarify, let $a_{i j l}$ and $a_{i k h}$ denote the number of visits by patient *i* to physician *j* in hospital *l* and physician *k* in hospital *h*, respectively. The weighted edge between physician *j* in hospital *l* and *k* in hospital *h* across *n* shared-patients can then be represented as $e_{j l k h}=\sum _{i=1}^{n} \sqrt{a_{i j l} a_{i k h}}$. We use the method introduced in Bynum et al. (2007) to assign physicians to hospitals and then compute weighted edges between hospitals by aggregating the physicians’ edge weights over the physician dyads spanning each pair of hospitals. That is, we compute the weighted edge between hospital *l* and *h* as $E_{l h}=\sum _{j l k h=1}^{m} e_{j l k h}$ with the summation over all *m* pairs of physicians bridging hospitals *l* and *h*.

**Fig. 5:**
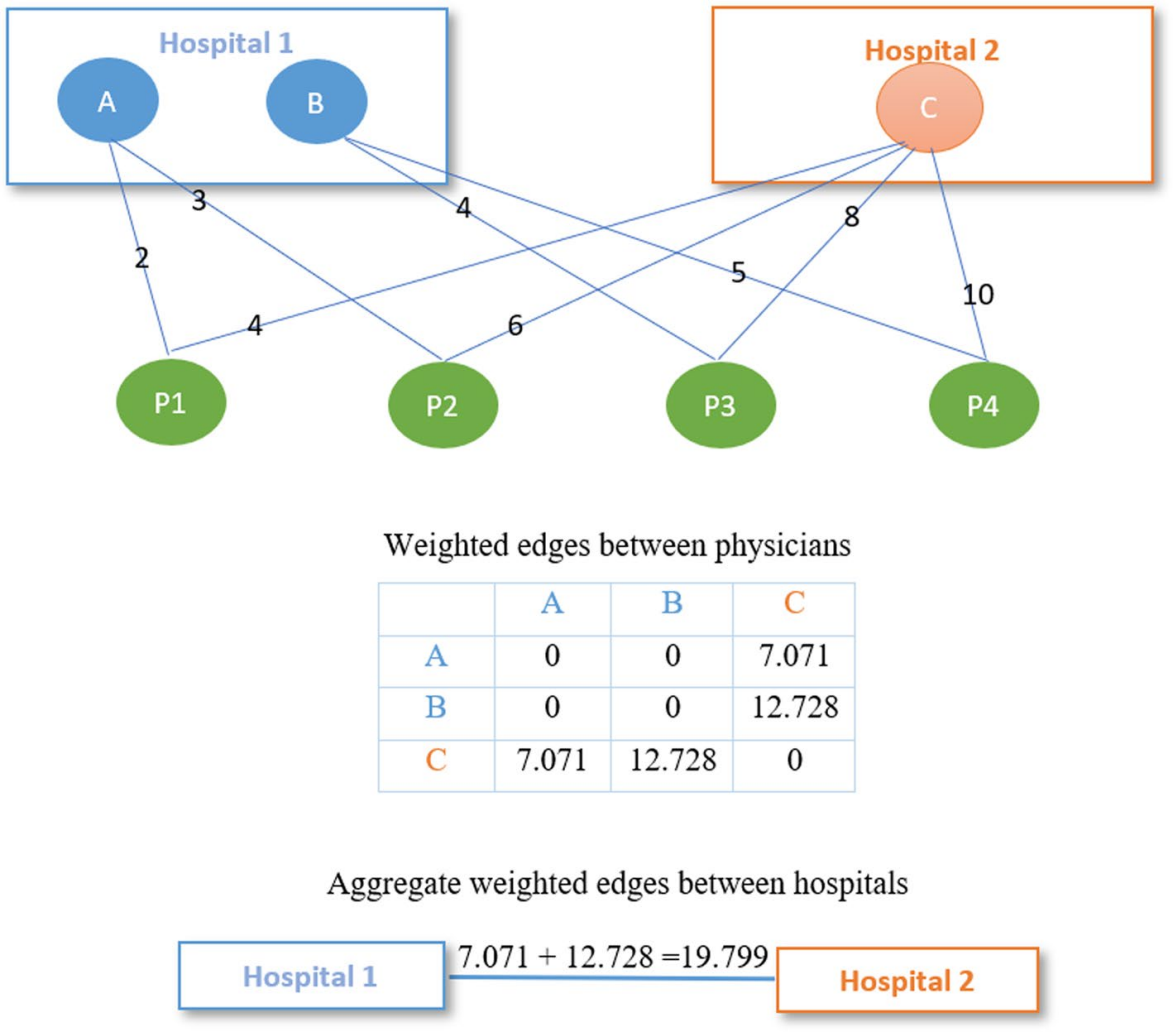
Depiction on the construction of patient sharing hospital network. Note: Nodes A, B and C represent physicians assigned to hospital 1 and 2, respectively. P1 to P4 represent patients shared by these physicians. Numbers on the ties between physicians and patients represent the number of visits by each patient to each physician

We use an example to depict the computation of weighted edges between physicians and aggregated weighted edges between hospitals (Fig. 5). As demonstrated above, the weighted edge between physicians is computed by summing the geometric means of a patient’s number of visits to each physician in a dyad across all patients. In the example in Fig. 5, the weighted edge between physician A and C is $\sqrt{2 \times 4}+\sqrt{3 \times 6} = 7.071$ and the weighted edge between physician B and C is $\sqrt{4 \times 8}+\sqrt{5 \times 10} = 12.728$. The weighted edge between hospitals is then computed by aggregating the edges over the physician dyads bridging each pair of hospitals. Because 2 pairs of physicians (A, C and B, C) span hospitals 1 and 2, the weighted edge between hospital 1 and 2 is $7.071 + 12.728=19.799$. The resulting undirected weighted hospital network matrix is row normalized to form the *W* used in the application of models (1) and (2) to these data.

We focus on a sub-group of hospitals in the network that are equipped with a robotic surgery system and that conducted more than 5 prostatectomies per year to study the association between the peer hospital adoption of robotic surgery and patients’ prostatectomy time to discharge post-surgery. Medicare health insurance claims data from 2016 is used to build the patient-sharing hospital network and evaluate hospital covariates while the 2017 Medicare data is used to evaluate all other patient outcomes and covariates. In our analysis, we include patient’s age, disability, whether receiving a robotic surgery and the Charlson Comorbidity Index (Charlson et al. 1994) as covariates. Because most patients ( 96%) have a Charlson Comorbidity Index of 0, we converted the Charlson Comorbidity index to a binary variable using 0 as the threshold; i.e., patients with comorbidity versus patients without comorbidity. For hospital-level covariates, we include the number of beds, percentage of robotic prostatectomy and number of peer hospitals in the network. For the outcome, patient’s prostatectomy time to discharge post-surgery, we use a log plus 1 transformation of the data to reduce skewness. In addition, we standardize all continuous covariates to ensure estimates and operating characteristics of these variables are on the same scale. The cohort contains 45 hospitals and 1,306 patients with the network density $d=0.779$ ($1 / \lambda _{\max }=1$ and $1 / \lambda _{\min }=-1.660$).

We report the posterior median estimators and $95 \%$ equal-tailed credible intervals of $\rho$, $\alpha$ and other parameters in Table 5. In addition, we compute the Deviance Information Criterion (DIC) (Spiegelhalter et al. 2002) for model comparison due to its data-determined evaluation of the effective degrees-of-freedom of the model to penalize our Bayesian hierarchical models for model complexity and thus guard against over-fitting when comparing the extended hierarchical network autocorrelation model (model (2)) to its base model counterpart in which $\alpha =0$ (model (1)).

**Table 5:** Estimates, credible interval and DIC for model (1) and (2)

Predictors and Key Model Parameters	Estimate (95% Equal-tailed Credible Interval)	
	Model 1	Model 2
Intercept	0.971(0.922, 1.021)	0.973(0.921, 1.029)
Whether done by robotic surgery	$-0.164 (-0.214, -0.114)$	$-0.164 (-0.214, -0.114)$
Age	0.052(0.029, 0.076)	0.052(0.029, 0.076)
Disability	0.184(0.112, 0.256)	0.184(0.112, 0.257)
Charlson Comorbidity Index	0.145(0.046, 0.244)	0.144(0.045, 0.243)
Beds	$-0.015 (-0.039, 0.008)$	$-0.016 (-0.045, 0.007)$
Percentage of robotic prostatectomy	$0.004 (-0.018, 0.027)$	$0.006 (-0.016, 0.029)$
Number of peer hospitals	$0.010 (-0.013, 0.032)$	$0.007 (-0.017, 0.031)$
$\rho$	$-0.048 (-1.164, 0.771)$	$-0.525 (-1.481, 0.804)$
$\alpha$	NA	$1.355 (-2.539, 4.168)$
$\sigma ^{2}$ (residual variance)	0.127(0.117, 0.137)	0.127(0.117, 0.137)
$\omega ^{2}$ (variance of random effects)	1.673E$-$04 (3.465E$-$07, 1.772E$-$03)	2.150E$-$04 (2.663E$-$07, 2.173E$-$03)
DIC	1018.878	1016.843

The results are for the prior $p(\rho ) \propto 1$, $1 / \lambda _{\min }<\rho <1 / \lambda _{\max }$; similar findings are observed for the other non-informative priors. Numbers are rounded to 3 decimal places

Table 3 finds similar results for all parameters other than the peer effect parameters $\rho$ and $\alpha$ between the two models. For instance, robotic surgery is associated with shorter hospital stays, while age, disability, and the Charlson Comorbidity index are associated with longer hospital stays. Comparing the two models, we observe a change in the estimated value of $\rho$. With the inclusion of $\alpha$, $\hat{\rho }$ changes from $-$0.048 ($-$1.164, 0.771) to $-$0.525 ($-$1.481, 0.804). Though not statistically significant (the credible interval of $\rho$ overlaps 0), the negative value of $\hat{\rho }$ indicates that peer hospitals’ adoption of robotic surgery indirectly associates with shorter hospital stays of patients. The interpretation of $\alpha$ is similar to that of a coefficient at a higher level than the observation level in a standard hierarchical regression model and $\hat{\alpha }=1.355$ ($-$2.539, 4.168) suggests that peer hospitals’ propensity to adopt robotic surgery is directly associated with longer patient hospital stays, although the result is far from statistically significant. The wide credible intervals for both $\rho$ and $\alpha$ overlap 0, revealing that with a very large network density (i.e., 0.779), the information in the data about $\rho$ and $\alpha$ is limited (much more so than if density were lower). Because $\rho$ and $\alpha$ are not significantly different from 0, it is not surprising that the DICs of the two models are very close in value. The DIC of model (2) is slightly smaller than the DIC for model (1), suggesting that model (2) fits the data better, even after accounting for its increased complexity.

## Discussion

In this paper, we developed two hierarchical network autocorrelation models to study the direct and indirect peer effects of actors at a higher level of the hierarchical data structure. The novel contributions include the exploration of both direct and indirect peer effects among higher-level actors and the impact of peer actor behavior on an observation-level outcome. In addition, we proposed a Bayesian approach for estimation and compared the performance of the resulting estimators under different prior distributions for the model parameters, with particular focus on $\rho$, to gain insights into the sensitivity of the posterior distribution and associated inferences to the prior.

For model (2), we set $W_{1}=W_{2}=W$ because only a single source of network relationship information was available. However, $W_{1}$ and $W_{2}$ may be different matrices representing different types of connections between actors. For example, a shared patient hospital network is representative of many other potential networks, including shared-physician and shared-specialist networks (a hospital-hospital network based on healthcare professionals working at multiple facilities). Using a shared-physician network for $W_{2}$ might have yielded more informative results in our robotic surgery study. This is a limitation of our study. Therefore, the performance of our models under different specifications of *W* is an important topic for future research.

In this paper, we focused on models that assume peer effects act on the outcomes themselves. However, the peer effect could instead act on the error term. In social network analysis, models with both autocorrelated outcome and autocorrelated error terms have also been considered (Anselin 1988; O’Malley and Marsden 2008). These considerations suggest a series of additional avenues of further work (e.g., the indirect effect could be modeled using an autocorrelated outcome term while the direct effect could be modeled through an autocorrelated error term). While our focus was on continuous outcomes, another direction for further research is to generalize the hierarchical and extended hierarchical network autocorrelation models to non-continuous outcomes such as binary, count and rate outcomes.

Homophily (the tendency for relationships to form between actors having similar attributes) may be a confounder to peer-influence. In the context of a shared patient network, homophily may be represented by predictors that quantify the similarity of hospital characteristics, in which case the associated regression coefficients capture the magnitude of homophily in relation to a given outcome. Because we are not modeling the network itself, the inferences on the coefficients of such predictors would not test the hypothesis that homophily is driving the formation of the network itself. Rather, these coefficients would test the hypothesis that patients of hospitals with more similar values of these predictors have more similar outcomes. An appealing extension of the involvement of homophily terms is to allow interaction effects between the similarity of hospital-level predictors (i.e., hospital-level homophily measures) and the magnitude of peer effects. This extension is a direction for future research in the vein of O’Malley et al. (2020), which considered a related term in a longitudinal linear peer-effect model. Although our simulation study confirmed that our models are estimable, further study of the relationship between network features and the precision of estimation of peer effects is warranted.

Our model and methodological development were applied to data from an observational study of the diffusion of robotic surgery on the quality of patient outcomes. Although our findings were inconclusive with the credible intervals of $\rho$ and $\alpha$ overlapping 0, a consequence of the densely connected network resulting in their being a modest amount of information in the data about $\rho$ and $\alpha$, in general our models have the potential to be widely applied and to reveal important scientific findings regarding the direct and indirect impact of the adoption of a health technology by peer hospitals on the outcomes of patients at the focal hospital, from which important policy recommendations may be derived.

## Supplementary Information

Supplementary Material 1.

## Data Availability

The data used for the motivating analyses contain patient identifiable information and so cannot be made available. However, template R code for performing the simulations (which can be easily adapted to analyze a real data set) have been uploaded to the paper’s GitHub site at: https://github.com/chen918/HNAM.

## Appendix

### Hierarchical network autocorrelation model

As discussed in the main text, the conditional posterior of $\rho$ does not have a well-known form for direct sampling. Therefore, we use the Metropolis-Hastings algorithm with an independent candidate generating function. To approximate the target distribution, we firstly approximate *ln*|*A*| using the quadratic polynomial approximation by a Taylor series expansion at $\rho =0$ (Dittrich et al. 2017):

$$\begin{aligned} |A|=\prod \left( 1-\rho \lambda _{i}\right), \end{aligned}$$

where $\lambda _{i}(i=1, \ldots, g)$ are eigenvalues of *W* (Ord 1975) and $\ln |A|=\sum \ln \left( 1-\rho \lambda _{i}\right)$. By a second-order Taylor series expansion:

$$\begin{aligned} \ln |A| \approx \sum -\rho \lambda _{i}-\rho ^{2} \lambda _{i}^{2} / 2=\sum -\rho ^{2} \lambda _{i}^{2} / 2 \text{ as } \sum \lambda _{i}={\text {tr}}(W)=0. \end{aligned}$$

That is, $|A| \approx \exp \left( \sum -\rho \lambda _{i}-\frac{\rho ^{2} \lambda _{i}^{2}}{2}\right) =\exp \left( \sum -\frac{\rho ^{2} \lambda _{i}^{2}}{2}\right)$. Therefore,

5 $$\begin{aligned} &p\left( \rho \mid \beta, \omega ^{2}, \delta, \sigma ^{2}, \theta, Y\right) \\ &\quad \propto \exp \left( \sum -\frac{\rho ^{2} \lambda _{i}^{2}}{2}\right) \exp \left( -\frac{(A \delta -X \beta )^{T}(A \delta -X \beta )}{2 \omega ^{2}}\right) \\ &\quad\sim TN\left( \mu ^{*}, V^{*}\right), 1 / \lambda _{\min }<\rho <1 / \lambda _{\text{ max } } \end{aligned}$$

where, $TN\left( \mu ^{*}, V^{*}\right)$ is the truncated normal distribution candidate generating distribution with mean $\mu ^{*}$ and variance $V^{*}$:

$$\begin{aligned} \begin{aligned} \mu ^{*}&=\frac{\delta ^{T} W^{T}(\delta -X \beta )}{\omega ^{2} \sum \lambda _{i}{ }^{2}+\delta ^{T} W^{T} W \delta } \\ V^{*}&=\frac{\omega ^{2}}{\omega ^{2} \sum \lambda _{i}{ }^{2}+\delta ^{T} W^{T} W \delta } \end{aligned} \end{aligned}$$

The acceptance ratio $\gamma$ for a Metropolis-Hastings algorithm with an independence candidate generating function is:

$$\begin{aligned} \gamma =\min \left( 1, \frac{\pi \left( \rho _{p}\right) TN\left( \rho _{c}, \mu ^{*}, V^{*}\right) }{\pi \left( \rho _{c}\right) TN\left( \rho _{p}, \mu ^{*}, V^{*}\right) }\right) \end{aligned}$$

where $\rho _{c}$ is the current state of $\rho$ and $\rho _{p}$ is the proposed state of $\rho$, is randomly generated from the candidate generating distribution with

$$\begin{aligned} \pi (\rho )=|A| \exp \left( -\frac{(A \delta -X \beta )^{T}(A \delta -X \beta )}{2 \omega ^{2}}\right). \end{aligned}$$

To perform a Metropolis-Hastings step we randomly draw a random variable *u* from *U*(0, 1) and accept the proposed state if $u \le \gamma$, otherwise rejecting the proposed state and staying at current state (i.e., if $u>\gamma$).

Similarly, for $p(\rho ) \propto 1,-1<\rho <1$, the resulting candidate generating distribution of $\rho$ is $TN\left( \mu ^{*}, V^{*}\right)$, $-1<\rho <1$. For

$$\begin{aligned} p(\rho ) \propto \frac{1}{\left( 1 / \lambda _{\max }-\rho \right) \left( \rho -1 / \lambda _{\min }\right) }, \end{aligned}$$

the conditional posterior of $\rho$ is:

$$\begin{aligned} p\left( \rho \mid \beta, \omega ^{2}, \delta, \sigma ^{2}, \theta, Y\right) \propto |A| \exp \left( -\frac{(A \delta -X \beta )^{T}(A \delta -X \beta )}{2 \omega ^{2}}\right) \frac{1}{\left( 1 / \lambda _{\max }-\rho \right) \left( \rho -1 / \lambda _{\min }\right) } \end{aligned}$$

The above distribution does not have a well-known closed-form that can be used for directly sampling. Therefore, we use the same candidate generating distribution introduced in Eq. (5) to sample $\rho$.

The conditional posterior of $\delta$ is:

$$\begin{aligned} & p\left( \delta \mid \beta, \omega ^{2}, \rho, \sigma ^{2}, \theta, Y\right) \propto \exp \left( -\frac{(A \delta -X \beta )^{T}(A \delta -X \beta )}{2 \omega ^{2}}-\frac{(Y-Z \theta -B \delta )^{T}(Y-Z \theta -B \delta )}{2 \sigma ^{2}}\right) \\ &\quad =\exp \left( \frac{\frac{\sigma ^{2}}{w^{2}}(A \delta -X \beta )^{T}(A \delta -X \beta )+(Y-Z \theta -B \delta )^{T}(Y-Z \theta -B \delta )}{-2 \sigma ^{2}}\right). \end{aligned}$$

Writing $H=\frac{\sigma }{\omega } A$, so that

$$\begin{aligned} =\exp \left( \frac{\left( H \delta -\frac{\sigma }{\omega } X \beta \right) ^{T}\left( H \delta -\frac{\sigma }{\omega } X \beta \right) +(Y-Z \theta -B \delta )^{T}(Y-Z \theta -B \delta )}{-2 \sigma ^{2}}\right). \end{aligned}$$

we then let $\Delta =\frac{\sigma }{\omega } X \beta$ to obtain

$$\begin{aligned} \left( H \delta -\frac{\sigma }{\omega } X \beta \right) ^{T}\left( H \delta -\frac{\sigma }{\omega } X \beta \right) =\left( \delta ^{T} H^{T}-\Delta ^{T}\right) (H \delta -\Delta ). \end{aligned}$$

Letting $M=Y-Z \theta$ we then obtain

$$\begin{aligned} (Y-Z \theta -B \delta )^{T}(Y-Z \theta -B \delta )=(M-B \delta )^{T}(M-B \delta )=\left( M^{T}-\delta ^{T} B^{T}\right) (M-B \delta ) \end{aligned}$$

and

$$\begin{aligned} (H \delta -\Delta )^{T}(H \delta -\Delta )+(M-B \delta )^{T}(M-B \delta ) \propto \delta ^{T}\left( H^{T} H+B^{T} B\right) \delta -2\left( \Delta ^{T} H+M^{T} B\right) \delta \end{aligned}$$

Finally, let $D=H^{T} H+B^{T} B, C=\Delta ^{T} H+M^{T} B$ so that

$$\begin{aligned} \delta ^{T} D \delta -2 C \delta \propto \left( \delta -D^{-1} C^{T}\right) ^{T} D\left( \delta -D^{-1} C^{T}\right). \end{aligned}$$

Hence,

$$\begin{aligned} p\left( \delta \mid \beta, \omega ^{2}, \rho, \sigma ^{2}, \theta, Y\right) \sim \textrm{N}\left( D^{-1} C^{T}, \sigma ^{2} D^{-1}\right). \end{aligned}$$

### Extended hierarchical network autocorrelation model

The likelihood function of the model (2) is:

$$\begin{aligned} f\left( Y \mid \theta, \delta, \sigma ^{2}, \alpha \right) =\left( 2 \pi \sigma ^{2}\right) ^{-N / 2} \exp \left( -\frac{(Y-Z \theta -B(\delta +\alpha W \delta ))^{T}(Y-Z \theta -B(\delta +\alpha W \delta ))}{2 \sigma ^{2}}\right). \end{aligned}$$

We assign a flat prior for $\alpha$, that is $p(\alpha ) \propto 1$ and the prior distributions for the other parameters align with these in model (1).

Letting $K=Y-Z \theta -B \delta$, the conditional posterior of $\alpha$ can be expressed as:

$$\begin{aligned} p\left( \alpha \mid \beta, \omega ^{2}, \rho, \sigma ^{2}, \theta, Y, \delta \right) \sim N\left( \frac{\delta ^{T} W^{T} B^{T} K}{\delta ^{T} W^{T} B^{T} B W \delta }, \frac{\sigma ^{2}}{\delta ^{T} W^{T} B^{T} B W \delta }\right) \end{aligned}$$

Moreover, the conditional posterior of $\delta$ is:

$$\begin{aligned} & p\left( \delta \mid \beta, \omega ^{2}, \rho, \sigma ^{2}, \theta, Y, \alpha \right) \\ &\quad \propto \exp \left( -\frac{(A \delta -X \beta )^{T}(A \delta -X \beta )}{2 \omega ^{2}}\right. \\ &\quad \left. -\frac{(Y-Z \theta -B(\delta +\alpha W \delta ))^{T}(Y-Z \theta -B(\delta +\alpha W \delta ))}{2 \sigma ^{2}}\right) \end{aligned}$$

Let $G=B\left[ I_{g}+\alpha W\right], D=H^{T} H+G^{T} G, C=\Delta ^{T} H+M^{T} G$. Therefore, $p\left( \delta \mid \beta, \omega ^{2}, \rho, \sigma ^{2}, \theta, Y, \alpha \right) \propto N\left( D^{-1} C^{T}, \sigma ^{2} D^{-1}\right)$. Similar to the model (1), the conditional posterior of $\theta, \sigma ^{2}, \omega ^{2}, \beta$ and $\rho$ are:

$$\begin{aligned} &P\left( \theta \mid \delta, \sigma ^{2}, \rho, \omega ^{2}, \beta, Y, \alpha \right) \sim N\left( \left( Z^{T} Z\right) ^{-1} Z^{T}(Y-B(\delta +\alpha W \delta )),\left( Z^{T} Z\right) ^{-1} \sigma ^{2}\right) \\ &P\left( \sigma ^{2} \mid \delta, \theta, \beta, \omega ^{2}, \rho, Y, \alpha \right) \sim I G\left( \frac{N-1}{2}, \frac{(Y-Z \theta -B(\delta +\alpha W \delta ))^{T}(Y-Z \theta -B(\delta +\alpha W \delta ))}{2}\right) \\ &P\left( \omega ^{2} \mid \delta, \theta, \beta, \sigma ^{2}, \rho, Y, \alpha \right) \sim I G\left( \frac{g-1}{2}, \frac{(A \delta -X \beta )^{T}(A \delta -X \beta )}{2}\right) \\ &P\left( \beta \mid \delta, \sigma ^{2}, \rho, \omega ^{2}, \theta, Y, \alpha \right) \sim N\left( \left( X^{T} X\right) ^{-1} X^{T} A \delta, \left( X^{T} X\right) ^{-1} \omega ^{2}\right) \\ &p\left( \rho \mid \beta, \omega ^{2}, \delta, \sigma ^{2}, \theta, Y, \alpha \right) \propto |A| \exp \left( -\frac{(A \delta -X \beta )^{T}(A \delta -X \beta )}{2 \omega ^{2}}\right) p(\rho ) \end{aligned}$$

To sample $\rho$, we use the same approach used for the model in (1).

### Half Cauchy prior for $\omega$

Let $\eta =\delta /\xi$ and $\sigma _{\eta }=\omega /|\xi |$, so that we can write model (1) as

$$\begin{aligned} &Y=Z \theta +B \xi \eta +\varepsilon \\ &\eta =\rho W \eta +\frac{X \beta }{\xi }+\frac{\tau }{\xi } \\ &\varepsilon \sim N\left( 0, \sigma ^{2} I_{N}\right) \\ &\frac{\tau }{\xi } \sim N\left( 0, \sigma _{\eta }^{2} I_{g}\right) \end{aligned}$$

The likelihood function can be expressed as:

$$\begin{aligned} f\left( Y \mid \theta, \xi, \eta, \sigma ^{2}\right) =\left( 2 \pi \sigma ^{2}\right) ^{-N / 2} \exp \left( -\frac{(Y-Z \theta -B \xi \eta )^{T}(Y-Z \theta -B \xi \eta )}{2 \sigma ^{2}}\right) \end{aligned}$$

while the prior distribution of $\eta$ is:

$$\begin{aligned} p\left( \eta \mid \beta, \sigma _{\eta }^{2}, \rho, \xi \right) =|A|\left( 2 \pi \sigma _{\eta }^{2}\right) ^{-\frac{g}{2}} \exp \left( -\frac{\left( A \eta -\frac{X \beta }{\xi }\right) ^{T}\left( A \eta -\frac{X \beta }{\xi }\right) }{2 \sigma _{\eta }^{2}}\right). \end{aligned}$$

We assign these priors for $\xi$ and $\sigma _{\eta }^{2}: \xi \sim N\left( 0, \phi ^{2}\right), \sigma _{\eta }^{2} \sim IG(0.5,0.5)$. Given the expected range of $\omega$ is under 100, we set $\phi =25$ and when $\phi \rightarrow \infty$, the half Cauchy prior for $\omega$ becomes a uniform prior for $\omega$ (Gelman 2006). We also assign uniform prior distributions on $\theta, \beta, \sigma$ and $p(\rho ) \propto 1$, $1 / \lambda _{\min }<\rho <1 / \lambda _{\max }$.

The conditional posteriors for the model parameters under the model in (1) follow below:

$$\begin{aligned} &P\left( \sigma ^{2} \mid \xi, \eta, \theta, \beta, \sigma _{\eta }^{2}, \rho, Y\right) \sim IG\left( \frac{N-1}{2}, \frac{(Y-Z \theta -B \xi \eta )^{T}(Y-Z \theta -B \xi \eta )}{2}\right) \\ &P\left( \theta \mid \xi, \eta, \sigma ^{2}, \rho, \sigma _{\eta }^{2}, \beta, Y\right) \sim N\left( \left( Z^{T} Z\right) ^{-1} Z^{T}(Y-B \xi \eta ),\left( Z^{T} Z\right) ^{-1} \sigma ^{2}\right) \\ &p\left( \rho \mid \beta, \sigma _{\eta }^{2}, \xi, \eta, \sigma ^{2}, \theta, Y\right) \propto \exp \left( \sum -\frac{\rho ^{2} \lambda _{i}^{2}}{2}\right) \exp \left( -\frac{(A \xi \eta -X \beta )^{T}(A \xi \eta -X \beta )}{2 \xi ^{2} \sigma _{\eta }^{2}}\right) \\ &P\left( \beta \mid \xi, \eta, \sigma ^{2}, \rho, \sigma _{\eta }^{2}, \theta, Y\right) \sim N\left( \left( X^{T} X\right) ^{-1} X^{T} A \xi \eta, \left( X^{T} X\right) ^{-1} \xi ^{2} \sigma _{\eta }^{2}\right) \\& p\left( \eta \mid \beta, \xi, \sigma _{\eta }^{2}, \rho, \sigma ^{2}, \theta, Y\right) \\ &\quad\propto \exp \left( -\frac{(A \xi \eta -X \beta )^{T}(A \xi \eta -X \beta )}{2 \xi ^{2} \sigma _{\eta }^{2}}\right) \exp \left( -\frac{(Y-Z \theta -B \xi \eta )^{T}(Y-Z \theta -B \xi \eta )}{2 \sigma ^{2}}\right) \\ &p\left( \xi \mid \beta, \eta, \sigma _{\eta }^{2}, \rho, \sigma ^{2}, \theta, Y\right) \propto \exp \left( -\frac{\xi ^{2}}{2 \phi ^{2}}\right) \exp \left( -\frac{(A \xi \eta -X \beta )^{T}(A \xi \eta -X \beta )}{2 \xi ^{2} \sigma _{\eta }^{2}}\right) \\ &\quad\times \exp \left( -\frac{(Y-Z \theta -B \xi \eta )^{T}(Y-Z \theta -B \xi \eta )}{2 \sigma ^{2}}\right) \\ &P\left( \sigma _{\eta }^{2} \mid \xi, \eta, \theta, \beta, \sigma ^{2}, \rho, Y\right) \propto \left( \sigma _{\eta }^{2}\right) ^{-\frac{g}{2}} \exp \left( -\frac{(A \xi \eta -X \beta )^{T}(A \xi \eta -X \beta )}{2 \xi ^{2} \sigma _{\eta }^{2}}\right) \left( \sigma _{\eta }^{2}\right) ^{-\frac{1}{2}-1} \exp \left( -\frac{1}{2 \sigma _{\eta }^{2}}\right) \\ &\quad\sim IG\left( \frac{g+1}{2}, \frac{(A \xi \eta -X \beta )^{T}(A \xi \eta -X \beta )+\xi ^{2}}{2 \xi ^{2}}\right) \end{aligned}$$

In addition, the conditional posterior of $\eta$ is:

$$\begin{aligned} &p\left( \eta \mid \beta, \xi, \sigma _{\eta }^{2}, \rho, \sigma ^{2}, \theta, Y\right) \propto \exp \left( -\frac{(A \xi \eta -X \beta )^{T}(A \xi \eta -X \beta )}{2 \xi ^{2} \sigma _{\eta }^{2}}-\frac{(Y-Z \theta -B \xi \eta )^{T}(Y-Z \theta -B \xi \eta )}{2 \sigma ^{2}}\right) \\ &\quad =\exp \left( \frac{\frac{\sigma ^{2}}{\xi ^{2} \sigma _{\eta }^{2}}(A \xi \eta -X \beta )^{T}(A \xi \eta -X \beta )+(Y-Z \theta -B \xi \eta )^{T}(Y-Z \theta -B \xi \eta )}{-2 \sigma ^{2}}\right) \end{aligned}$$

Let

$$\begin{aligned} H=\frac{\sigma }{\xi \sigma _{\eta }} A =\exp \left( \frac{\left( H \xi \eta -\frac{\sigma }{\xi \sigma _{\eta }} X \beta \right) ^{T}\left( H \xi \eta -\frac{\sigma }{\xi \sigma _{\eta }} X \beta \right) +(Y-Z \theta -B \xi \eta )^{T}(Y-Z \theta -B \xi \eta )}{-2 \sigma ^{2}}\right) \end{aligned}$$

and $$\Delta =\frac{\sigma }{\xi \sigma _{\eta }} X \beta$$.

$$\begin{aligned} \left( H \xi \delta -\frac{\sigma }{\xi \sigma _{\eta }} X \beta \right) ^{T}\left( H \xi \delta -\frac{\sigma }{\xi \sigma _{\eta }} X \beta \right) =\left( \xi \delta ^{T} H^{T}-\Delta ^{T}\right) (\xi H \delta -\Delta ). \end{aligned}$$

Then letting $$M=Y-Z \theta$$,

$$\begin{aligned} (Y-Z \theta -B \xi \eta )^{T}(Y-Z \theta -B \xi \eta )=(M-B \xi \eta )^{T}(M-B \xi \eta )=\left( M^{T}-\xi \eta ^{T} B^{T}\right) (M-\xi B \eta ) \end{aligned}$$

and

$$\begin{aligned} (H \xi \eta -\Delta )^{T}(H \xi \eta -\Delta )+(M-B \xi \eta )^{T}(M-B \xi \eta ) \propto \xi ^{2} \eta ^{T}\left( +B^{T} B\right) \eta -2 \xi \left( \Delta ^{T} H+M^{T} B\right) \eta \end{aligned}$$

Finally, let $$D=H^{T} H+B^{T} B, C=\Delta ^{T} H+M^{T} B$$

$$\begin{aligned} \xi ^{2} \eta ^{T} D \eta -2 \xi C \eta \propto \left( \xi \eta -D^{-1} C^{T}\right) ^{T} D\left( \xi \eta -D^{-1} C^{T}\right) \end{aligned}$$

Hence,

$$\begin{aligned} p\left( \eta \mid \beta, \xi, \sigma _{\eta }^{2}, \rho, \sigma ^{2}, \theta, Y\right) \sim \textrm{N}\left( D^{-1} C^{T} / \xi, \sigma ^{2} D^{-1} / \xi ^{2}\right). \end{aligned}$$

Therefore, the conditional posterior of $\xi$ is:

$$\begin{aligned} &p\left( \xi \mid \beta, \eta, \sigma _{\eta }^{2}, \rho, \sigma ^{2}, \theta, Y\right) \propto \exp \left( -\frac{\xi ^{2}}{2 \phi ^{2}}\right) \exp \left( -\frac{(A \xi \eta -X \beta )^{T}(A \xi \eta -X \beta )}{2 \xi ^{2} \sigma _{\eta }^{2}}\right) \\ &\quad\times \exp \left( -\frac{(Y-Z \theta -B \xi \eta )^{T}(Y-Z \theta -B \xi \eta )}{2 \sigma ^{2}}\right) \\ \end{aligned}$$

To sample $\xi$ from its conditional posterior distribution, we used following candidate generating function:

$$\begin{aligned} &p\left( \xi \mid \beta, \eta, \sigma _{\eta }^{2}, \rho, \sigma ^{2}, \theta, Y\right) \sim N\left( \mu _{\xi }, V_{\xi }\right) \\ &\mu _{\xi } =\frac{\eta ^{T} B^{T}(Y-Z \theta )}{\eta ^{T} B^{T} B \eta +\sigma ^{2} / \phi ^{2}} \\ &V_{\xi } =\frac{\sigma ^{2}}{\eta ^{T} B^{T} B \eta +\sigma ^{2} / \phi ^{2}} \end{aligned}$$

The conditional posterior of $\rho$ is:

$$\begin{aligned} p\left( \rho \mid \beta, \sigma _{\eta }^{2}, \xi, \eta, \sigma ^{2}, \theta, Y\right) \propto \exp \left( -\sum \frac{\rho ^{2} \lambda _{i}^{2}}{2}\right) \exp \left( -\frac{(A \xi \eta -X \beta )^{T}(A \xi \eta -X \beta )}{2 \xi ^{2} \sigma _{\eta }^{2}}\right). \end{aligned}$$

As previously discussed, the candidate generate function of $\rho$ is:

$$\begin{aligned} &TN\left( \mu ^{*}, V^{*}\right) \\ &\mu ^{*}=\frac{\eta ^{T} W^{T}(\eta -X \beta / \xi )}{\sigma _{\eta }^{2} \sum \lambda _{i}{ }^{2}+\eta ^{T} W^{T} W \eta } \\ &V^{*}=\frac{\sigma _{\eta }^{2}}{\sigma _{\eta }^{2} \sum \lambda _{i}{ }^{2}+\eta ^{T} W^{T} W \eta } \end{aligned}$$

In the above, we used model (1) to show the derivation of the half Cauchy prior for $\omega$. A similar construction applies for (2).
