# Supplementary material for: Bayesian hierarchical network autocorrelation models for estimating direct and indirect effects of peer hospitals on outcomes of hospitalized patients
Source: Appl Netw Sci. 2024 Jun 14;9(1):24. doi: 10.1007/s41109-024-00627-1 (PMC11636997; doi:10.1007/s41109-024-00627-1)
Supplement: Supplementary file 1 — Supplementary Material 1. [file 41109_2024_627_MOESM1_ESM.pdf]

Bayesian Hierarchical Network Autocorrelation Models for Estimating Direct and Indirect Effects of Peer Hospitals on Outcomes of Hospitalized Patients

Guanqing Chen and A. James O'Malley

List of Tables …………………………………………………………………………….. 1

S1 ……………………………………………………………………………………………… 2
Bias and mean squared error and 95% coverage rate of ρ for the model 1 when the parameter space of ρ in the prior is restricted to $(1/\lambda_{min}, 1/\lambda_{max})$.

S2 ……………………………………………………………………………………………… 3
Bias and mean squared error and 95% coverage rate of ρ for the model 1 when the parameter space of ρ in the prior is restricted to $(-1,1)$.

S3 ……………………………………………………………………………………………… 4
Bias and mean squared error and 95% coverage rate of ρ for the model 1 when $p(\rho) \propto \frac{1}{(1/\lambda_{max}-\rho)(\rho-1/\lambda_{min})}$.

S4 ……………………………………………………………………………………………… 5
Bias, mean squared error and 95% coverage rate of ρ and α for the model 2 when the parameter space of ρ in the prior is restricted to $(1/\lambda_{min}, 1/\lambda_{max})$.

S5 ……………………………………………………………………………………………… 6
Bias, mean squared error and 95% coverage rate of ρ and α for the model 2 when the parameter space of ρ in the prior is restricted to $(-1,1)$.

S6 ……………………………………………………………………………………………… 7
Bias, mean squared error and 95% coverage rate of ρ and α for the model 2 when $p(\rho) \propto \frac{1}{(1/\lambda_{max}-\rho)(\rho-1/\lambda_{min})}$.

Table 1. Bias, mean squared error (MSE) and 95% coverage rate (Rate) of ρ using $U(1/\lambda_{min}, 1/\lambda_{max})$ prior.

Network Density		$\rho = -0.5$	$\rho = -0.2$	$\rho = 0$	$\rho = 0.2$	$\rho = 0.5$
0.2	Bias	0.034	0.026	0.017	0.007	-0.011
	MSE	0.026	0.028	0.029	0.029	0.025
	Rate	0.944	0.94	0.946	0.956	0.946
0.4	Bias	0.020	-0.019	0.010	0.010	-0.053
	MSE	0.069	0.071	0.077	0.069	0.058
	Rate	0.96	0.948	0.954	0.952	0.958
0.6	Bias	0.044	0.043	0.023	-0.057	-0.131
	MSE	0.145	0.151	0.152	0.120	0.108
	Rate	0.972	0.966	0.958	0.972	0.96
0.8	Bias	0.025	-0.091	-0.133	-0.172	-0.298
	MSE	0.346	0.290	0.272	0.270	0.263
	Rate	0.962	0.97	0.97	0.97	0.962

Note: The results are rounded to 3 decimal places.

Table S2. Bias, mean squared error (MSE) and 95% coverage rate (Rate) of ρ using $U(-1,1)$ prior.

Network Density		$\rho = -0.5$	$\rho = -0.2$	$\rho = 0$	$\rho = 0.2$	$\rho = 0.5$
0.2	Bias	0.023	0.024	0.006	0.007	-0.011
	MSE	*0.025*	*0.029*	*0.027*	*0.029*	*0.025*
	Rate	0.958	0.946	0.964	0.956	0.946
0.4	Bias	0.062	0.028	0.002	-0.019	-0.063
	MSE	*0.062*	*0.074*	*0.077*	*0.071*	*0.058*
	Rate	0.96	0.964	0.944	0.956	0.962
0.6	Bias	0.151	0.062	0.007	-0.024	-0.152
	MSE	*0.127*	*0.115*	*0.126*	*0.104*	*0.120*
	Rate	0.97	0.974	0.958	0.976	0.948
0.8	Bias	0.241	0.123	0.020	-0.102	-0.274
	MSE	*0.171*	*0.162*	*0.147*	*0.141*	*0.186*
	Rate	0.988	0.978	0.986	0.988	0.966

Note: The results are rounded to 3 decimal places.

Table S3. Bias, mean squared error (MSE) and 95% coverage rate (Rate) of ρ using the transformed uniform prior that is proportional to $\frac{1}{(1/\lambda_{max}-\rho)(\rho-1/\lambda_{min})}$ *when ρ is the subject of the distribution.*

Network Density		$\rho = -0.5$	$\rho = -0.2$	$\rho = 0$	$\rho = 0.2$	$\rho = 0.5$
0.2	Bias	0.033	0.035	0.037	0.043	0.054
	MSE	*0.028*	*0.031*	*0.033*	*0.035*	*0.035*
	Rate	0.942	0.934	0.930	0.924	0.916
0.4	Bias	0.048	0.068	0.086	0.101	0.074
	MSE	*0.088*	*0.103*	*0.109*	*0.107*	*0.068*
	Rate	0.946	0.934	0.924	0.906	0.906
0.6	Bias	0.148	0.173	0.170	0.151	0.067
	MSE	*0.266*	*0.265*	*0.233*	*0.186*	*0.104*
	Rate	0.920	0.912	0.916	0.920	0.944
0.8	Bias	0.312	0.283	0.233	0.165	0.026
	MSE	*0.591*	*0.475*	*0.377*	*0.278*	*0.161*
	Rate	0.912	0.918	0.926	0.934	0.944

Note: The results are rounded to 3 decimal places.

Table S4. Bias, mean squared error (MSE) and 95% coverage rate (Rate) of ρ and α using $U(1/\lambda_{min}, 1/\lambda_{max})$ prior for ρ.

Network Density		$\rho = -0.5$		$\rho = -0.2$		$\rho = 0$		$\rho = 0.2$		$\rho = 0.5$	
		$\alpha = -2$	$\alpha = 2$	$\alpha = -2$	$\alpha = 2$	$\alpha = -2$	$\alpha = 2$	$\alpha = -2$	$\alpha = 2$	$\alpha = -2$	$\alpha = 2$
$d = 0.2$	Bias	0.045	0.07	0.005	0.024	-0.005	0.01	-0.049	0.002	-0.082	-0.061
	MSE	0.049	0.068	0.052	0.059	0.06	0.053	0.063	0.049	0.062	0.046
	Rate	0.962	0.95	0.976	0.958	0.962	0.962	0.956	0.952	0.958	0.926
	Bias	-0.009	-0.021	0.002	-0.011	0.006	-0.004	0.02	-0.005	0.04	0.016
	MSE	0.012	0.007	0.012	0.007	0.011	0.006	0.011	0.007	0.016	0.008
	Rate	0.974	0.938	0.962	0.942	0.97	0.958	0.97	0.946	0.94	0.948
$d = 0.4$	Bias	0.019	0.067	-0.073	-0.017	-0.185	-0.105	-0.295	-0.164	-0.516	-0.236
	MSE	0.286	0.306	0.259	0.212	0.277	0.232	0.314	0.204	0.478	0.178
	Rate	0.96	0.97	0.978	0.98	0.984	0.972	0.96	0.952	0.942	0.928
	Bias	0.027	-0.045	0.09	0.019	0.173	0.056	0.255	0.091	0.449	0.135
	MSE	0.234	0.142	0.224	0.098	0.266	0.098	0.301	0.081	0.461	0.078
	Rate	0.944	0.966	0.96	0.972	0.956	0.974	0.954	0.958	0.894	0.934

Note: The results in blue font represent the results of ρ while the results in black font represent the results of α. The results are rounded to 3 decimal places.

Table S5. Bias, mean squared error (MSE) and 95% coverage rate (Rate) of ρ and α using $U(-1,1)$ prior for ρ.

Network Density		$\rho = -0.5$		$\rho = -0.2$		$\rho = 0$		$\rho = 0.2$		$\rho = 0.5$	
		$\alpha = -2$	$\alpha = 2$	$\alpha = -2$	$\alpha = 2$	$\alpha = -2$	$\alpha = 2$	$\alpha = -2$	$\alpha = 2$	$\alpha = -2$	$\alpha = 2$
$d = 0.2$	Bias	0.054	0.062	-0.001	0.049	0	0.009	-0.044	0.022	-0.054	-0.029
	MSE	0.045	0.05	0.054	0.057	0.053	0.05	0.065	0.055	0.059	0.037
	Rate	0.968	0.964	0.964	0.966	0.978	0.96	0.96	0.932	0.94	0.958
	Bias	-0.017	-0.012	0.006	-0.01	0.009	-0.004	0.017	-0.008	0.026	0.002
	MSE	0.012	0.006	0.012	0.008	0.011	0.007	0.014	0.008	0.011	0.007
	Rate	0.954	0.964	0.968	0.956	0.956	0.956	0.956	0.946	0.948	0.962
$d = 0.4$	Bias	0.234	0.364	0.056	0.134	-0.057	0.021	-0.147	-0.123	-0.367	-0.243
	MSE	0.171	0.239	0.129	0.145	0.125	0.125	0.133	0.135	0.249	0.172
	Rate	0.982	0.984	0.994	0.984	0.986	0.984	0.986	0.98	0.952	0.914
	Bias	-0.168	-0.239	-0.022	-0.095	0.069	-0.026	0.132	0.083	0.293	0.121
	MSE	0.125	0.151	0.102	0.093	0.111	0.068	0.132	0.073	0.207	0.072
	Rate	0.962	0.968	0.99	0.978	0.974	0.978	0.972	0.968	0.96	0.956

Note: The results in blue font represent the results of ρ while the results in black font represent the results of α. The results are rounded to 3 decimal places.

Table S6. Bias, mean squared error (MSE) and 95% coverage rate (Rate) of ρ using the transformed uniform prior that is proportional to $\frac{1}{(1/\lambda_{max}-\rho)(\rho-1/\lambda_{min})}$ *when ρ is the subject of the distribution .*

Network Density		$\rho = -0.5$		$\rho = -0.2$		$\rho = 0$		$\rho = 0.2$		$\rho = 0.5$	
		$\alpha = -2$	$\alpha = 2$	$\alpha = -2$	$\alpha = 2$	$\alpha = -2$	$\alpha = 2$	$\alpha = -2$	$\alpha = 2$	$\alpha = -2$	$\alpha = 2$
$d = 0.2$	Bias	0.046	0.065	0.033	0.075	0.036	0.068	0.046	0.039	0.026	0.031
	MSE	0.058	0.073	0.069	0.079	0.088	0.069	0.077	0.071	0.076	0.043
	Rate	0.954	0.942	0.95	0.94	0.934	0.934	0.95	0.912	0.91	0.918
	Bias	-0.009	-0.017	-0.004	-0.021	-0.002	-0.013	0.004	-0.018	0.005	-0.011
	MSE	0.013	0.008	0.011	0.01	0.015	0.009	0.01	0.01	0.01	0.009
	Rate	0.966	0.936	0.962	0.948	0.942	0.96	0.964	0.932	0.926	0.964
$d = 0.4$	Bias	0.135	0.376	0.15	0.246	0.028	0.17	-0.036	0.079	-0.13	-0.043
	MSE	0.442	0.635	0.405	0.47	0.398	0.294	0.407	0.223	0.361	0.119
	Rate	0.926	0.918	0.95	0.91	0.926	0.936	0.928	0.91	0.912	0.94
	Bias	-0.029	-0.238	-0.038	-0.166	0.043	-0.122	0.122	-0.036	0.178	0.027
	MSE	0.269	0.295	0.258	0.221	0.293	0.149	0.353	0.108	0.353	0.069
	Rate	0.946	0.928	0.956	0.916	0.936	0.958	0.926	0.952	0.92	0.95

Note: The results in blue font represent the results of ρ while the results in black font represent the results of α. The results are rounded to 3 decimal places.
